# The relationship of C-Reactive Protein to Albumin Ratio and interval debulking surgery outcome after neoadjuvant chemotherapy in ovarian cancer patients

**DOI:** 10.1016/j.clinsp.2024.100469

**Published:** 2024-08-03

**Authors:** Yi Zheng, Shuyu Liu, Mengran Chang, Caizhi Wang, Yu Zhou

**Affiliations:** Department of Obstetrics and Gynecology, The First Affiliated Hospital of Bengbu Medical College, Anhui Province, PR China

**Keywords:** C-Reactive Protein, Albumin, Ovarian Cancer, Interval Debulking Surgery, Neoadjuvant Chemotherapy

## Abstract

**Objective:**

To investigate the relationship between the changes of C-reactive protein to Albumin Ratio (CAR) levels and Interval Debulking Surgery (IDS) outcome after Neoadjuvant Chemotherapy (NAC) in ovarian cancer patients.

**Methods:**

A nested case-control study for 209 patients with ovarian cancer who received NAC-IDS therapy from the First Affiliated Hospital of Bengbu Medical College between 2015‒2021 was conducted. Demographic data, laboratory indicators, and imaging examinations were collected. The outcome was regarded as optimal IDS in this study. Univariate and multivariate logistic regression analyses were performed to assess the relationship of CAR before NAC, CAR after NAC and ∆CAR with optimal IDS. The authors also performed the subgroup analysis based on menopausal state.

**Results:**

The end time of follow-up was January 24, 2022. A total of 156 patients had been treated with optimal IDS, and 53 with suboptimal IDS. After adjusting age, body mass index, menopausal state, NAC drug, peritoneal perfusion and CAR before NAC, the result showed that CAR after NAC (Odds Ratio [OR = 3.48], 95% Confidence Interval [95% CI 1.28‒9.48], p = 0.015) and ∆CAR (OR = 0.29, 95% CI 0.11‒0.78, p = 0.015) were associated with optimal IDS, respectively. Additionally, the authors found a significant correlation between CAR after NAC and optimal IDS (OR = 3.16, 95% CI 1.07‒9.35, p = 0.038), and ∆CAR and optimal IDS (OR = 0.32, 95% CI 0.11‒0.94, p = 0.038) among ovarian cancer patients with menopause.

**Conclusion:**

CAR after NAC and ∆CAR were independent prognostic markers of optimal interval debulking surgery for ovarian cancer patients.

## Highlights

• Interval Debulking Surgery (IDS) treats ovarian cancer after neoadjuvant chemotherapy.
• Relation between changes of C-reactive protein to Albumin Ratio (CAR) and IDS outcome.
• CAR was an independent prognostic marker of optimal IDS for ovarian cancer patients.

## Introduction

Ovarian cancer is considered to be one of the most common gynecological malignancies, with more than 300,000 new cases and 200,000 deaths globally in 2020, threatening to women's life and health.^1^^,^^2^ Early ovarian cancer has no specific clinical symptoms, so most patients are diagnosed at advanced stages.^3^ The standard treatment for ovarian cancer contains optimal Primary Debulking Surgery (PDS) and adjuvant chemotherapy.^4^ Recently, there has been increasing evidence that Interval Debulking Surgery (IDS) After Neoadjuvant Chemotherapy (NAC) has been considered as an alternative treatment strategy for ovarian cancer, with lower postoperative complications, surgical complexity, and residual lesions compared to PDS.^4^^,^^5^ For patients undergoing IDS treatment, the postoperative residual lesion is the key factor affecting the survival of patients with ovarian cancer.^6^ Therefore, it is necessary to predict the residual status of the lesions after IDS, which may assist clinicians in evaluating the difficulty of surgery, choosing appropriate treatment ways and improving the prognosis of patients.

Previous studies have found a close link between ovarian cancer and inflammation.^7^^,^^8^ Therefore, the identification of inflammation-related biomarkers is of great interest in the prognosis of oncology. Recently, the C-reactive protein to Albumin Ratio (CAR), consisting of C-Reactive Protein (CRP) and albumin, is considered to be an important marker of inflammation.^9^ Liu Y, et al., have investigated the prognostic value of the preoperative CAR in ovarian cancer, indicating that the CAP was associated with poor prognosis of ovarian cancer patients and has a superior prognostic ability.^10^ In addition, a number of studies have expounded that preoperative Carbohydrate Antigen 125 (CA125) levels at different time points are related to surgical outcomes.^11^^,^^12^ The changes of serum CA125 after NAC were associated with residual lesions after IDS in patients with advanced epithelial ovarian cancer, and were also an independent predictor of satisfactory interval debulking surgery.^6^ In the study of Gülseren V, they also showed that the dynamic change in neutrophil-to-lymphocyte ratio values was related to the likelihood of suboptimal surgery in advanced-stage ovarian cancer patients who undergo IDS after NAC.^13^ These studies also suggested that dynamic changes of inflammatory markers play an important role in predicting surgical outcomes. However, to our knowledge, few studies have explored the predictive value of CAR at different time points in IDS outcomes.

Herein, this study considered ovarian cancer patients undergoing NAC-IDS, and aimed to analyze the effect of CAR before NAC, CAR after NAC and CAR dynamic changes on surgical outcomes.

## Methods

### Study design and data sources

**Fig. 1:**
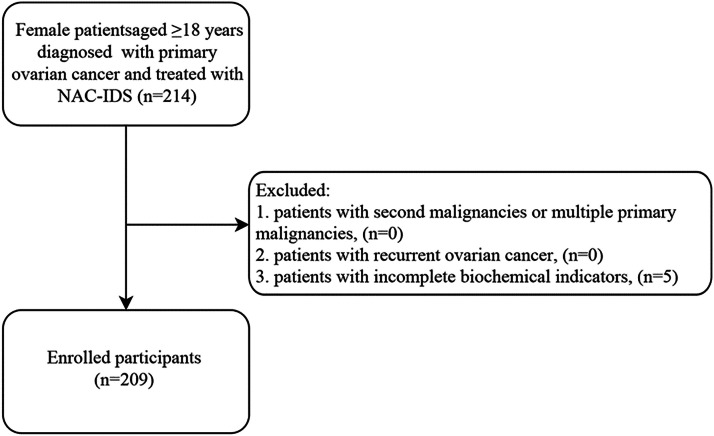
Flow chart of study population selection.

The authors designed a nested case-control study and selected patients who were diagnosed with ovarian cancer from the First Affiliated Hospital of Bengbu Medical College between 2015 and 2021. This observational study was performed based on the STROBE Statement. The inclusion criteria are as follows: (1) Female patients were ≥ 18 years of age; (2) Patients were pathologically diagnosed as primary ovarian cancer; (3) Patients received NAC-IDS therapy. Patients were excluded when they met one of the following criteria: (1) Patients had second malignancies or multiple primary malignancies; (2) Patients had recurrent ovarian cancer; (3) Patients had incomplete biochemical indicators. Finally, 209 patients with ovarian cancer were enrolled in this study (Fig. 1). The study was approved by the ethics committee of the First Affiliated Hospital of Bengbu Medical College (2022KY037). The study was conducted in accordance with the Declaration of Helsinki.

### Outcome and follow-up

The outcome was regarded as optimal IDS in this study. Optimal IDS was defined as the maximum diameter of residual tumor ≤1 cm. The end time of follow-up was January 24, 2022.

### Data collection

Patients' information was collected and analyzed, demographic characteristics included age, Body Mass Index (BMI, kg/m^2^), menopausal state, family history of ovarian cancer, family history of other cancer, hypertension, inflammation; Laboratory examination contained hemoglobin (g/L), Red Blood Cell (RBC), White Blood Cell Count (WBC), Platelet (PLT, 10^9^/L), neutrophil count (10^9^/L), lymphocyte count (10^9^/L), monocyte count (10^9^/L), eosinophils count (10^9^/L), basophils count (10^9^/L), Neutrophil to Lymphocyte Ratio (NLR), Platelet to Lymphocyte Ratio (PLR), Red blood cell Distribution Width (RDW, %), Mean Platelet Volume (MPV, fL), Alanine Transaminase (ALT, U/L), Aspartate Aminotransferase (AST, U/L), Gamma-Glutamyltransferase (GGT, U/L), Alkaline Phosphatase (ALP, U/L), Total Bilirubin (TBIL, μmoL/L), Direct Bilirubin (DBIL, μmoL/L), Indirect Bilirubin (IBIL, μmoL/L), Total Protein (TP, g/L), globin (GLB, g/L), Albumin to Globin Ratio (AGR), Blood Urea Nitrogen (BUN, mmoL/L), creatinine (μmoL/L), uric acid (μmoL/L), Carcinoembryonic Antigen (CEA, ng/mL), CA199 (IU/mL), CA125 (IU/mL), CA153 (IU/mL), Alpha Fetoprotein (AFP, IU/mL), Albumin (ALB) before NAC (g/L), ALB after NAC (g/L), C-Reactive Protein (CRP) before NAC (mg/L), CRP after NAC (mg/L), CAR before NAC, CAR after NAC, ∆CAR. Treatment: NAC drug, peritoneal perfusion, cycles of NAC. Imaging examination: International Federation of Gynecology and Obstetrics (FIGO) staging, grade staging and histology. CAR was defined as C-reactive protein to albumin ratio, and the authors recorded CAR value before NAC and after NAC. ∆CAR was calculated as CAR before NAC-CAR after NAC.

### Statistical analysis

Continuous variables were evaluated by mean ± Standard Deviation (SD) or median and quartile [M (Q1, Q3)], and Student's *t*-test or Mann-Whitney *U* test was used for comparison between groups. Categorical variables were evaluated by the number of cases and composition ratio [n (%)], and comparison between groups adopted the Chi-Square test or Fisher's exact test.

Univariate and multivariate logistic regression analyses were performed to assess the relationship of CAR before NAC, CAR after NAC and ∆CAR with optimal IDS. Odds Ratio (OR) and 95% Confidence Interval (95% CI) were calculated in this study. Subsequently, the authors performed the subgroup analysis based on the menopausal state. In the present study, these missing variables were interpolated, and sensitivity analysis was performed (Supplementary Table 1). SAS 9.4 (SAS Institute Inc., Cary, NC, USA) was used for statistical analysis; p < 0.05 was considered statistically significant.

## Results

### Baseline characteristics

209 patients were enrolled in the study. The mean age of the patients was 58.71±9.37 years. The median cycle of NAC was 2. Table 1 presents the clinical characteristics and laboratory parameters. 156 patients had been treated with optimal IDS, and 53 with suboptimal IDS. The authors compared the characteristics’ differences between the optimal IDS group and the suboptimal IDS group. As shown in Table 1, there were no statistical differences in the distribution of most variables, including age, BMI, menopausal state, family history of ovarian cancer, hypertension, hemoglobin, RBC, WBC, PLT, NLR, PLR, RDW, MPV, ALT, AST, GGT, NAC drug, peritoneal perfusion, grade staging and histology between the two groups (p > 0.05).

**Table 1:** General characteristics of all patients.

Variables	Total (n = 209)	Optimal IDS (n = 156)	Suboptimal IDS (n = 53)	p
Age, years, Mean ± SD	58.71 ± 9.37	58.88 ± 9.07	58.21 ± 10.28	0.654
Height, cm, Mean ± SD	157.87 ± 4.03	158.03 ± 3.81	157.38 ± 4.63	0.308
Weight, kg, Mean ± SD	59.35 ± 8.27	59.38 ± 8.53	59.27 ± 7.56	0.935
BMI, kg/m^2^, Mean ± SD	23.80 ± 3.08	23.76 ± 3.16	23.93 ± 2.87	0.730
Menopausal state, n (%)				0.444
Non-menopause	51 (24.40)	36 (23.08)	15 (28.30)	
Menopause	158 (75.60)	120 (76.92)	38 (71.70)	
Family history of ovarian cancer, n (%)				0.105
No	204 (97.61)	154 (98.72)	50 (94.34)	
Yes	5 (2.39)	2 (1.28)	3 (5.66)	
Family history of other cancer, n (%)				0.863
No	21 (10.05)	16 (10.26)	5 (9.43)	
Yes	188 (89.95)	140 (89.74)	48 (90.57)	
Hypertension, n (%)				0.730
No	158 (75.60)	117 (75.00)	41 (77.36)	
Yes	51 (24.40)	39 (25.00)	12 (22.64)	
Inflammation, n (%)				0.297
No	198 (94.74)	146 (93.59)	52 (98.11)	
Yes	11 (5.26)	10 (6.41)	1 (1.89)	
Hemoglobin, g/L, Mean ± SD	121.23 ± 13.20	121.12 ± 13.54	121.55 ± 12.25	0.840
RBC, Mean ± SD	4.27 ± 0.44	4.25 ± 0.43	4.33 ± 0.46	0.222
WBC, Mean ± SD	7.46 ± 2.05	7.49 ± 2.06	7.37 ± 2.02	0.709
PLT, Mean ± SD	387.08 ± 117.92	389.56 ± 122.80	379.75 ± 102.94	0.602
Neutrophil count, 109/L, M (Q1, Q3)	5.17 (3.96, 6.59)	5.31 (4.01, 6.58)	4.85 (3.89, 6.67)	0.423
Lymphocyte count, 109/L, M (Q1, Q3)	1.40 (1.10, 1.81)	1.40 (1.11, 1.80)	1.45 (1.04, 1.82)	0.815
Monocyte count, 109/L, M (Q1, Q3)	0.50 (0.39, 0.62)	0.50 (0.38, 0.62)	0.51 (0.41, 0.61)	0.701
Eosinophils count, 109/L, M (Q1, Q3)	0.08 (0.04, 0.15)	0.08 (0.05, 0.15)	0.07 (0.04, 0.19)	0.495
Basophils count, 109/L, M (Q1, Q3)	0.01 (0.00, 0.02)	0.01 (0.00, 0.02)	0.01 (0.00, 0.02)	0.977
NLR, M (Q1, Q3)	3.73 (2.46, 4.99)	3.63 (2.53, 5.01)	4.02 (2.21, 4.85)	0.959
PLR, M (Q1, Q3)	260.00 (191.35, 359.46)	263.25 (192.10, 357.35)	253.79 (190.91, 365.38)	0.976
RDW, %, Mean ± SD	42.25 ± 3.09	42.04 ± 2.93	42.88 ± 3.47	0.085
MPV, fL, Mean ± SD	10.46 ± 1.11	10.45 ± 1.07	10.47 ± 1.23	0.931
ALT, U/L, M (Q1, Q3)	14.00 (12.00, 17.00)	14.00 (11.00, 17.00)	15.00 (13.00, 18.00)	0.079
AST, U/L, M (Q1, Q3)	21.00 (18.00, 28.00)	21.00 (19.00, 28.00)	23.00 (18.00, 31.00)	0.451
GGT, U/L, M (Q1, Q3)	15.00 (12.00, 22.00)	15.00 (12.00, 22.00)	16.00 (12.00, 23.00)	0.887
ALP, U/L, M (Q1, Q3)	69.00 (57.00, 82.00)	68.00 (56.00, 82.00)	70.00 (58.00, 81.00)	0.757
TBIL, μmoL/L, M (Q1, Q3)	7.20 (5.60, 9.20)	7.25 (5.60, 9.45)	7.20 (5.80, 9.10)	0.905
DBIL, μmoL/L, M (Q1, Q3)	2.50 (1.70, 3.40)	2.60 (1.90, 3.40)	2.10 (1.50, 3.40)	0.131
IBIL, μmoL/L, M (Q1, Q3)	4.60 (3.50, 6.50)	4.55 (3.25, 6.50)	4.90 (3.70, 6.20)	0.348
TP, g/L, Mean ± SD	69.98 ± 7.22	70.03 ± 6.94	69.81 ± 8.05	0.847
GLB, g/L, Mean ± SD	32.07 ± 6.75	31.89 ± 6.21	32.59 ± 8.21	0.568
AGR, Mean ± SD	1.24 ± 0.34	1.25 ± 0.32	1.22 ± 0.40	0.676
BUN, mmoL/L, M (Q1, Q3)	4.30 (3.30, 5.56)	4.26 (3.30, 5.51)	4.40 (3.37, 5.92)	0.710
Creatinine, μmoL/L, Mean ± SD	60.40 ± 11.65	60.24 ± 11.95	60.87 ± 10.81	0.737
Uric Acid, μmoL/L, M (Q1, Q3)	271.00 (234.00, 345.00)	267.50 (231.00, 345.00)	298.00 (240.00, 345.00)	0.520
CEA, ng/mL, M (Q1, Q3)	1.31 (0.82, 2.01)	1.29 (0.82, 1.95)	1.35 (0.96, 2.14)	0.223
CA199, IU/mL, M (Q1, Q3)	9.02 (3.59, 21.70)	8.90 (3.55, 20.79)	9.04 (3.60, 29.20)	0.551
CA125, IU/mL, M (Q1, Q3)	1000.00 (652.30, 1617.20)	1000.00 (611.50, 1437.40)	1000.00 (730.00, 1965.20)	0.348
CA153, IU/mL, M (Q1, Q3)	62.07 (28.10, 134.00)	64.82 (29.80, 132.70)	51.20 (23.10, 135.80)	0.533
AFP, IU/mL, M (Q1, Q3)	2.67 (1.85, 3.91)	2.52 (1.81, 3.96)	2.88 (2.07, 3.91)	0.471
NAC drug, n (%)				0.359
Paclitaxel and platinum	72 (34.45)	51 (32.69)	21 (39.62)	
Docetaxel and platinum	137 (65.55)	105 (67.31)	32 (60.38)	
Peritoneal perfusion				0.787
Yes	19 (9.09)	15 (9.62)	4 (7.55)	
No	190 (90.91)	141 (90.38)	49 (92.45)	
NAC Cycle, M (Q1, Q3)	2.00 (2.00, 3.00)	2.00 (2.00, 3.00)	2.00 (2.00, 3.00)	0.832
ALB before NAC, g/L, Mean ± SD	37.90 ± 4.57	38.13 ± 4.47	37.21 ± 4.84	0.206
ALB after NAC, g/L, Mean ± SD	41.74 ± 4.27	42.14 ± 3.84	40.55 ± 5.20	0.044
CRP before NAC, mg/L, M (Q1, Q3)	39.60 (13.20, 71.40)	39.95 (14.44, 91.35)	38.02 (10.50, 55.00)	0.333
CRP after NAC, mg/L, M (Q1, Q3)	2.40 (1.20, 5.50)	2.08 (1.20, 5.00)	3.20 (1.60, 9.00)	0.059
CAR before NAC, M (Q1, Q3)	1.00 (0.33, 2.14)	1.02 (0.36, 2.38)	0.88 (0.28, 1.49)	0.402
CAR after NAC, M (Q1, Q3)	0.06 (0.03, 0.14)	0.05 (0.03, 0.12)	0.07 (0.04, 0.20)	0.053
∆CAR, M (Q1, Q3)	0.90 (0.19, 1.94)	0.96 (0.22, 2.33)	0.56 (0.16, 1.39)	0.079
FIGO staging, n (%)				<0.001
Ⅰ	17 (8.13)	17 (10.90)	0 (0.00)	
Ⅱ	26 (12.44)	23 (14.74)	3 (5.66)	
Ⅲ	152 (72.73)	115 (73.72)	37 (69.81)	
Ⅳ	14 (6.70)	1 (0.64)	13 (24.53)	
Grade staging, n (%)				0.165
Well-differentiated	7 (3.35)	5 (3.21)	2 (3.77)	
Moderately-differentiated	11 (5.26)	10 (6.41)	1 (1.89)	
Intermediate between well-differentiated and moderately differentiated	13 (6.22)	8 (5.13)	5 (9.43)	
Poorly-differentiated	127 (60.77)	90 (57.69)	37 (69.81)	
Unknown	51 (24.40)	43 (27.56)	8 (15.09)	
Histology, n (%)				0.268
Serous cystadenocarcinoma	139 (66.51)	103 (66.03)	36 (67.92)	
Non-serous adenocarcinoma	52 (24.88)	37 (23.72)	15 (28.30)	
Unknown	18 (8.61)	16 (10.26)	2 (3.77)	

IDS, Interval Debulking Surgery; BMI, Body Mass Index; RBC, Red Blood Cell; WBC, White Blood Cell Count; PLT, Platelet; NLR, Neutrophil to Lymphocyte Ratio; PLR, Platelet to Lymphocyte Ratio; RDW, Red blood cell Distribution Width; MPV, Mean Platelet Volume; ALT, Alanine Transaminase; AST, Aspartate Aminotransferase; GGT, Gamma-Glutamyltransferase; ALP, Alkaline Phosphatase; TBIL, Total Bilirubin; DBIL, Direct Bilirubin; IBIL, Indirect Bilirubin; TP, Total Protein; GLB, Globin; AGR, Albumin to Globin Ratio; BUN, Blood Urea Nitrogen; CEA, Carcinoembryonic Antigen; CA199, Carbohydrate Antigen-199; CA125, Carbohydrate Antigen-125; CA153, Carbohydrate Antigen-153; AFP, Alpha Fetoprotein; ALB, Albumin; CAR, C-Reactive Protein to Albumin Ratio; NAC, Neoadjuvant Chemotherapy; CRP, C-Reactive Protein; FIGO, International Federation of Gynecology and Obstetrics; SD, Standard Deviation.

### The relationship of CAR before NAC, CAR after NAC and ∆CAR with optimal IDS

Table 2 indicates the relationship of CAR before NAC, CAR after NAC and ∆CAR with optimal IDS. In univariate analysis, CAR after NAC (Model 1: OR = 2.69, 95% CI 1.10‒6.60, p = 0.030) and ∆CAR (Model 1: OR = 0.80, 95% CI 0.65‒0.99, p = 0.046) were associated with optimal IDS, respectively. After adjusting age, BMI, menopausal state, NAC drug, and peritoneal perfusion, the result showed an association between CAR after NAC (Model 2: OR = 3.00, 95% CI 1.18‒7.63, p = 0.021) and ∆CAR (Model 2: OR = 0.79, 95% CI 0.63‒0.99, p = 0.037) with optimal IDS. After further adjusting age, BMI, menopausal state, NAC drug, peritoneal perfusion, and CAR before NAC, the relationship of CAR after NAC (Model 3: OR = 3.48, 95% CI 1.28‒9.48, p = 0.015) and ∆CAR (Model 3: OR = 0.29, 95% CI 0.11‒0.78, p = 0.015) with optimal IDS were still present. It is worth noting that as regards the correlation between CAR before NAC and optimal IDS, there was no significant difference (p > 0.05). In addition, since CAR is an indicator of inflammation, the authors performed a sensitivity analysis of inflammatory patients before and after deletion (Supplementary Table 2), the result showed that the association between CAR after NAC and ∆CAR with optimal IDS was robust.

**Table 2:** The relationship of CAR before NAC, CAR after NAC and ∆CAR with optimal IDS.

Variables	Model 1		Model 2		Model 3	
	OR (95% CI)	p	OR (95% CI)	p	OR (95% CI)	p
CAR before NAC	0.87 (0.70‒1.07)	0.175	0.86 (0.69‒1.06)	0.151	‒	‒
CAR after NAC	2.69 (1.10‒6.60)	0.030	3.00 (1.18‒7.63)	0.021	3.48 (1.28‒9.48)	0.015
∆CAR	0.80 (0.65‒0.99)	0.046	0.79 (0.63‒0.99)	0.037	0.29 (0.11‒0.78)	0.015

NAC, Neoadjuvant Chemotherapy; CAR, C-reactive protein to Albumin Ratio; IDS, Interval Debulking Surgery; OR, Odds Ratio; CI, Confidence Interval; Model 1, Not adjusted variables; Model 2, Adjusted age, body mass index, menopausal state, NAC drug and peritoneal perfusion; Model 3, adjusted age, body mass index, menopausal state, NAC drug, peritoneal perfusion and CAR before NAC.

### Subgroup analysis based on menopausal state

The authors also assessed the relationship of CAR before NAC, CAR after NAC and ∆CAR with optimal IDS based on the menopausal state of all populations. Table 3 displays the result of multivariate logistic regression analysis. After adjusting for some covariates, among ovarian cancer patients with menopause, the authors found a significant correlation between CAR after NAC and optimal IDS (OR = 3.16, 95% CI 1.07‒9.35, p = 0.038), and ∆CAR and optimal IDS (OR=0.32, 95% CI 0.11‒0.94, p = 0.038).

**Table 3:** Subgroup analysis base on menopausal state.

Variables	OR (95% CI)	p
Non-menopause (n = 51)		
CAR before NAC^a^	0.96 (0.65‒1.40)	0.820
CAR after NAC^b^	5.18 (0.21‒125.31)	0.311
∆CAR^b^	0.19 (0.01‒4.66)	0.311
Menopause (n = 158)		
CAR before NAC^a^	0.81 (0.61‒1.06)	0.129
CAR after NAC^b^	3.16 (1.07‒9.35)	0.038
∆CAR^b^	0.32 (0.11‒0.94)	0.038

NAC, Neoadjuvant Chemotherapy; CAR, C-reactive protein to Albumin Ratio; OR, Odds Ratio; CI, Confidence Interval.

a Adjusted age, body mass index, menopausal state, NAC drug and peritoneal perfusion.

b Adjusted age, body mass index, menopausal state, NAC drug, peritoneal perfusion and CAR before NAC.

## Discussion

This study evaluated data for 209 ovarian cancer patients who underwent NAC followed by IDS, and investigated the effect of CAR before NAC, CAR after NAC, and CAR dynamic changes on surgical outcomes. The present results indicated that CAR after NAC and ∆CAR were associated with the risk of optimal IDS, respectively.

Ovarian cancer was still the most common death from gynecological tumors. A recent study has suggested that the percentage of high-grade, advanced-stage ovarian cancer patients with complete response to chemotherapy, absence of recurrent disease, and lymphovascular space invasion were considered prognostic indicators for survival in patients with ovarian carcinoma.^14^ Several studies have described the clinical application of immunotherapy for ovarian cancer. Programmed cell Death 1 (PD-1)/Programmed Death-Ligand 1 (PD-L1) pathway mediates tumor immune escape, rendering it a promising target for immunotherapeutic interventions.^15^ In the study of Gutic, et al., it was found that PD-1/PD-L1 blockade therapy may serve as the primary approach for cancer immunotherapy, thereby potentially improving patient prognosis.^16^ However, the safety and efficacy of PD-1/PD-L1 blockade therapy necessitate further deliberation. It is well known that inflammation is closely related to the occurrence, development and metastasis of tumors.^17^ Tumor-induced inflammation can lead to DNA damage and micro-metastatic lesions, while the systemic inflammatory response may exacerbate patients’ malnutrition and promote tumor growth, invasion, angiogenesis and even metastasis.^18^^,^^19^ Previous studies have pointed out that inflammation plays a key role in ovarian cancer.^7^^,^^20^ Some inflammatory markers have been considered to be predictors of prognosis in patients with ovarian cancer, such as NLR,^21^ PLR,^22^ Glasgow Prognostic Score (GPS)/Modified Glasgow Prognostic Score (mGPS),^23^ and so on. CAR, was a combination of CRP and albumin, reflects the inflammatory state and nutritional state of cancer patients.^24^ In the study of Liu Y, et al., patients with high CAR had poor overall survival compared to patients with low CAR, and CAR was also shown to be independent prognostic factors for overall survival, which had a superior prognostic ability than GPS, mGPS and Prognostic Nutritional Index (PNI).^10^ A possible mechanism is explained as follows:^25^, ^26^, ^27^ cachexia is present in patients with advanced ovarian cancer, and cancer cachexia might be associated with systemic inflammation. Thus, hypoalbuminemia or elevated CRP has been found to be related to cancer cachexia.

However, previous studies only focused on the relationship of CAR and survival of patients with ovarian cancer, few studies assessed the effect of CAR at different time points on surgical outcomes for ovarian cancer patients undergoing NAC-IDS. In the current study, after adjusting age, BMI, menopausal state, NAC drug, peritoneal perfusion and CAR before NAC, the authors found that CAR after NAC was a risk factor of optimal IDS, and (OR = 3.48, 95% CI 1.28‒9.48) and ∆CAR was a protective factor for optimal IDS (OR = 0.29, 95% CI 0.11‒0.78). Especially for ovarian cancer patients with menopause, the correlation between CAR after NAC and optimal IDS (OR = 3.16, 95% CI 1.07‒9.35), and ∆CAR and optimal IDS (OR = 0.32, 95% CI 0.11‒0.94) were present. These results also indicated that CAR level may be useful in monitoring the postoperative outcome among ovarian cancer patients undergoing NAC-IDS. However, the mechanism of the relationship of CAR after NAC and ∆CAR with optimal IDS was still unclear. Further studies are needed to offer mechanisms underlying the correlation between CAR after NAC and ∆CAR with optimal IDS.

To the best of our knowledge, this is the first study to investigate the effect of CAR levels at different time points on postoperative outcomes in ovarian cancer patients undergoing NAC-IDS. This study considers the changes in the body's CAR levels during treatment and provides some reference for monitoring the efficacy of NAC and the timing of surgery for IDS. However, there are several limitations to this study. Firstly, due to the retrospective, observational nature of this study, the authors must acknowledge the existence of bias. Secondly, the optimal IDS reported in this study reflects only the last 6 years of IDS treatment for ovarian cancer at the present center, and differences in NAC across different centers may lead to different postoperative outcomes. Lastly, the present study focused only on surgical outcomes, and further prospective studies with a large sample will be required to explore the impact of CAR before NAC, CAR after NAC and CAR dynamic changes on the survival of ovarian cancer patients undergoing NAC-IDS.

## Conclusion

In short, the present study showed that CAR after NAC and ∆CAR were independent prognostic markers in ovarian cancer patients undergoing NAC-IDS, respectively. More studies are needed to offer mechanisms underlying the correlation between CAR after NAC and ∆CAR with optimal IDS.

## Abbreviations

CAR, C-Reactive protein to Albumin Ratio; IDS, Interval Debulking Surgery; PDS, Primary Debulking Surgery; NAC, Neoadjuvant Chemotherapy; CRP, C-Reactive Protein; CA125, Carbohydrate Antigen-125; BMI, Body Mass Index; RBC, Red Blood Cell; WBC, White Blood Cell Count; PLT, Platelet; NLR, Neutrophil to Lymphocyte Ratio; PLR, Platelet to Lymphocyte Ratio; RDW, Red blood cell Distribution Width; MPV, Mean Platelet Volume; ALT, Alanine Transaminase; AST, Aspartate Aminotransferase; GGT, Gamma-Glutamyltransferase; ALP, Alkaline Phosphatase; TBIL, Total Bilirubin; DBIL, Direct Bilirubin; IBIL, Indirect Bilirubin; TP, Total Protein; GLB, Globin; AGR, Albumin to Globin Ratio; BUN, Blood Urea Nitrogen; CEA, Carcinoembryonic Antigen; AFP, Alpha Fetoprotein; ALB, Albumin; FIGO, International Federation of Gynecology and Obstetrics; SD, Standard Deviation; OR, Odds Ratio; CI, Confidence Interval; GPS, Glasgow Prognostic Score; mGPS, Modified Glasgow Prognostic Score; PNI, Prognostic Nutritional Index.

## Declarations

### Ethics approval and consent to participate

The study was conducted in accordance with the Declaration of Helsinki. The study was approved by the ethics committee of the First Affiliated Hospital of Bengbu Medical College (2022KY037). The written informed consent was obtained from the participants.

### Consent for publication

Not applicable.

## Authors’ contributions

(1) Yi Zheng, Yu Zhou, conceiving and designing the study.

(2) Yi Zheng, Shuyu Liu, Mengran Chang, Caizhi Wang, collecting the data.

(3) Yi Zheng, Shuyu Liu, Mengran Chang, Caizhi Wang, analyzing and interpreting the data.

(4) Yi Zheng, writing the manuscript.

(5) Yu Zhou, Yi Zheng, providing critical revisions that are important for the intellectual content.

(6) Yi Zheng, Shuyu Liu, Mengran Chang, Caizhi Wang, Yu Zhou, approving the final version of the manuscript.

## Funding

This study was supported by the Science Research Project of Bengbu Medical College (2020byzd149).

## Declaration of competing interest

The authors declare no conflicts of interest.

## Data Availability

The datasets used and/or analyzed during the current study are available from the corresponding author on reasonable request.

## Appendix Supplementary materials

Image, application 1
