# Supplementary material for: The relationship of C-Reactive Protein to Albumin Ratio and interval debulking surgery outcome after neoadjuvant chemotherapy in ovarian cancer patients
Source: Clinics (Sao Paulo). 2024 Aug 3;79:100469. doi: 10.1016/j.clinsp.2024.100469 (PMC11345303; doi:10.1016/j.clinsp.2024.100469)
Supplement: Supplementary file 1 [file mmc1.docx]

**CLINICS-D-24-00188_Supplementary Material**

**Supplemental Table 1** The sensitivity analysis of missing data before and after interpolation.

| **Variables** | **Ratio of missing values (%)** | **Before the interpolation** | **After the interpolation** | **p** |
| --- | --- | --- | --- | --- |
| MPV, Mean ± SD | 1.91 | 10.47 ± 1.12 | 10.46 ± 1.11 | 0.903 |
| DBIL, M (Q1, Q3) | 1.91 | 2.50 (1.70, 3.40) | 2.50 (1.70, 3.40) | 0.954 |
| IBIL, M (Q1, Q3) | 1.91 | 4.60 (3.50, 6.50) | 4.60 (3.50, 6.50) | 0.966 |
| CEA, M (Q1, Q3) | 12.44 | 1.32 (0.83, 2.06) | 1.31 (0.82, 2.01) | 0.795 |
| CA199, M (Q1, Q3) | 13.40 | 9.04 (3.60, 21.67) | 9.02 (3.59, 21.70) | 0.951 |
| CA125, M (Q1, Q3) | 2.87 | 1000.00 (637.00, 1446.00) | 1000.00 (652.30, 1617.20) | 0.840 |
| AFP, M (Q1, Q3) | 14.35 | 2.67 (1.81, 3.91) | 2.67 (1.85, 3.91) | 0.888 |
| CA153, M (Q1, Q3) | 15.31 | 58.50 (24.80, 131.00) | 62.07 (28.10, 134.00) | 0.741 |

MPV, Mean Platelet Volume; DBIL, Direct Bilirubin; IBIL, Indirect Bilirubin; CEA, Carcinoembryonic Antigen; CA199, Carbohydrate Antigen-199; CA125, Carbohydrate Antigen-125; CA153, Carbohydrate Antigen-153; AFP, Alpha Fetoprotein.

**Supplemental Table 2** Sensitivity analysis of inflammatory patients before and after deletion.

| **Variables** | **Before deletion** | | **After deletion** | |
| --- | --- | --- | --- | --- |
|  | **OR (95% CI)** | **p** | **OR (95% CI)** | **p** |
| CAR before NAC^a^ | 0.80 (0.62‒1.05) | 0.105 | 0.84 (0.67‒1.04) | 0.102 |
| CAR after NAC^b^ | 3.48 (1.28‒9.48) | 0.015 | 3.92 (1.23‒12.45) | 0.021 |
| ∆CAR^b^ | 0.29 (0.11‒0.78) | 0.015 | 0.76 (0.61‒0.96) | 0.022 |

NAC, Neoadjuvant Chemotherapy; CAR, C-reactive protein to Albumin Ratio; OR, Odds Ratio; CI, Confidence Interval.

^a^ Adjusted age, body mass index, menopausal state, NAC drug and peritoneal perfusion.

^b^ Adjusted age, body mass index, menopausal state, NAC drug, peritoneal perfusion and CAR before NAC.
